# Dual effect of PEG-PE micelle over the oligomerization and fibrillation of human islet amyloid polypeptide

**DOI:** 10.1038/s41598-018-22820-w

**Published:** 2018-03-13

**Authors:** Xiaocui Fang, Maryam Yousaf, Qunxing Huang, Yanlian Yang, Chen Wang

**Affiliations:** 1 CAS Key Laboratory of Standardization and Measurement for Nanotechnology, CAS Key Laboratory of Biological Effects of Nanomaterials and Nanosafety, CAS Center for Excellence in Nanoscience, National Center for Nanoscience and Technology, Beijing, 100190 P. R. China; 2 Radiation Chemistry Laboratory, Department of Chemistry, University of Agriculture, Faisalabad, 38000 Pakistan

## Abstract

The oligomerization and fibrillation of human islet amyloid polypeptide (hIAPP) play a central role in the pathogenesis of type 2 diabetes. Strategies for remodelling the formation of hIAPP oligomers and fibrils have promising application potential in type 2 diabetes therapy. Herein, we demonstrated that PEG-PE micelle could inhibit hIAPP oligomerization and fibrillation through blocking the hydrophobic interaction and the conformational change from random coil to β-sheet structures of hIAPP. In addition, we also found that PEG-PE micelle could remodel the preformed hIAPP fibrils allowing the formation of short fibrils and co-aggregates. Taken together, PEG-PE micelle could rescue hIAPP-induced cytotoxicity by decreasing the content of hIAPP oligomers and fibrils that are related to the oxidative stress and cell membrane permeability. This study could be beneficial for the design and development of antiamyloidogenic agents.

## Introduction

The deposition of insoluble amyloid aggregates, formed due to misfolding of proteins and peptides, is involved in the pathogenesis of many amyloidogenic diseases including Parkinson’s disease (PD), Huntington’s disease (HD), Alzheimer’s disease (AD), mad cow disease, and type 2 diabetes (T2DM)^1–3^. The aggregation of human islet amyloid polypeptide (hIAPP) is one of the common representative examples because of its rapid aggregation dynamics. hIAPP is a 37-residue peptide synthesized and co-secreted along with insulin in pancreatic β-cells^4^. hIAPP shows the propensity to aggregate from its normally soluble and functional states into insoluble and β-sheet-rich amyloid^5–7^. hIAPP aggregates are the main component of pancreatic amyloid deposits, one of the characteristic pathological features of T2DM^5,8,9^. Extensive studies have shown that the deposition of hIAPP amyloid is associated with pancreatic β-cell dysfunction and loss of β-cell mass, which is the main cause of T2DM pathogenesis^6,10^. In this regard, inhibitors targeting hIAPP aggregates hold great application potential.

Although hIAPP adopts various conformations *i.e*., from monomer, dimer and soluble oligomer to protofibril and fibril during its aggregation process, it has been generally accepted that amyloid oligomers have greater toxicity compared to monomers or fibrils. Early hIAPP inhibitors have focused on the insoluble fibrils as they were thought to be the primary pathogenic species due to their prevalence in the pancreatic amyloid deposits^9^. A number of inhibitors, such as peptides^11^, coordination compounds^12,13^, small molecules^14^, nanoparticles^15^, dendritic polymers^16,17^ and macromolecules^18^, have been developed to bind with hIAPP and to remodel its assembly. These molecular binding agents have been shown to disrupt the fibrillation of hIAPP and to rescue hIAPP-induced cytotoxicity. In recent years, the attention has been shifted towards the early stage of hIAPP fibrillation *i.e*., soluble oligomers and protofibrils as they were suggested to be the primary toxic species involved in β-cell dysfunction and cell death in T2DM. However, none of the hIAPP commercial inhibitor has been approved clinically for T2DM therapy, due to the efficiency of these inhibitors usually vary from case to case and is also condition-dependent. The current clinical treatment for T2DM is a continuous subcutaneous insulin infusion, but the repeated insulin injection in the same site can cause local reactions of subcutaneous scleroma, fat atrophy and ache, which limits its widespread application. Therefore, it is highly desirable to develop more effective candidates for hIAPP inhibitors.

PEG-PE molecule consists of hydrophilic polyethylene glycol (PEG) and hydrophobic phosphatidylethanolamine (PE). The polymeric micelle comprised of amphiphilic PEG-PE molecules is a promising nano-sized system for both chemotherapeutic drugs and peptides because of their good biocompatibility and safety^19–23^. Our previous studies have demonstrated that PEG-PE micelle could assist non-native insulin refolding into its native states and inhibit insulin aggregation through blocking hydrophobic interactions of dithiothreitol (DTT)-denatured insulin A and B chains^20^. This prompted us whether PEG-PE micelle could remodel hIAPP assembly and rescue hIAPP-induced cytotoxicity.

Herein, we chose two hIAPP peptides (hIAPP_1__-__37_ and hIAPP_8__-__37_) and investigated the inhibitory effect of PEG-PE micelle on both hIAPP_1__-__37_ and hIAPP_8__-__37_ aggregation. These results demonstrated that PEG-PE micelle not only inhibited the oligomerization and fibrillation of hIAPP_1__-__37_ and hIAPP_8__-__37_, but also rescued hIAPP_1__-__37_- and hIAPP_8__-__37_-mediated β-cell death. We further investigated the mechanism underlying the alleviated cytotoxicity by PEG-PE micelle, and found that PEG-PE micelle reduced hIAPP_1__-__37_- and hIAPP_8__-__37_-induced intracellular oxidative stress and cell membrane permeability. In addition, we also evaluated the inhibitory effect of PEG-PE micelle over the preformed hIAPP_1__-__37_ and hIAPP_8__-__37_ fibrils, and found that PEG-PE micelle could rescue hIAPP_1__-__37_ and hIAPP_8__-__37_ fibrils-induced cytotoxicity through remodelling the entangled long fibrils into short fibrils and co-aggregates. This work will broaden the application of PEG-PE as an antiamyloidogenic agent.

## Results

### The inhibitory effect of PEG-PE micelle on hIAPP_1__-__37_ and hIAPP_8__-__37_ fibrillogenesis

**Figure 1:**
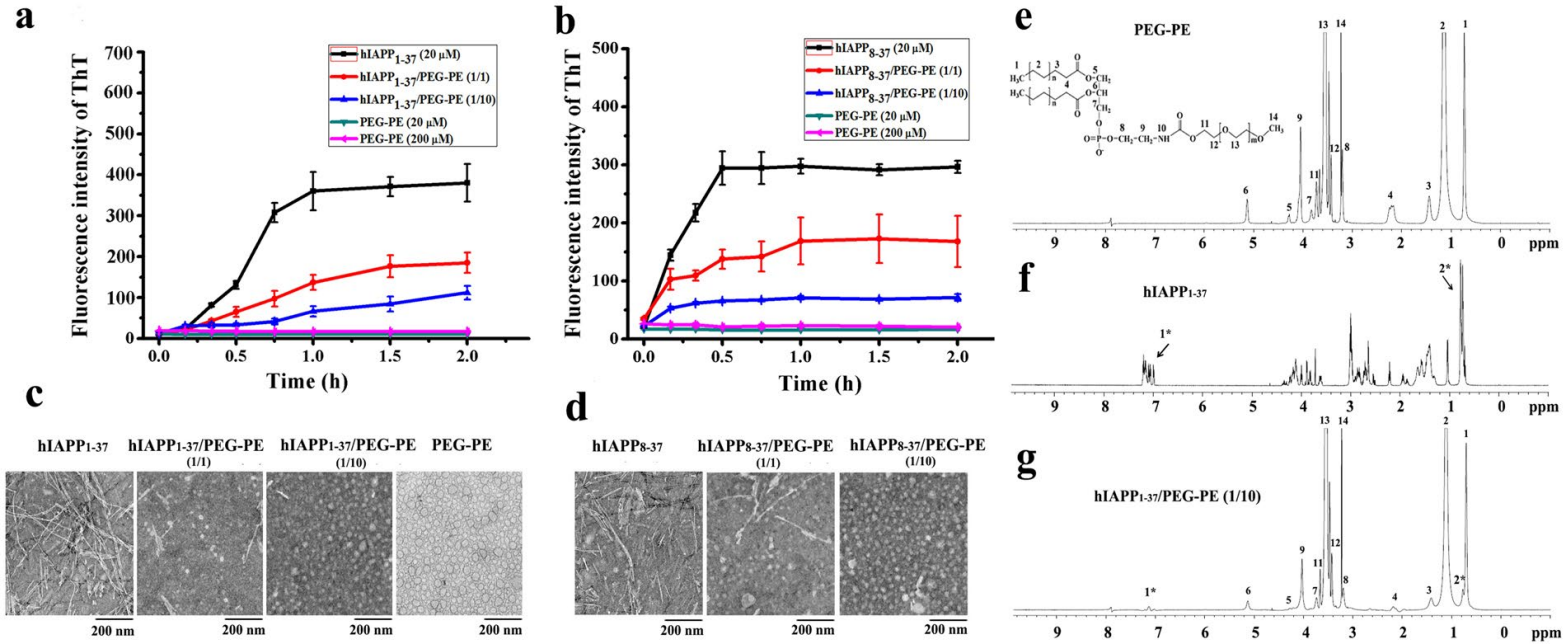
Antiamyloidogenic effect of PEG-PE micelles on hIAPP_1-37_ and hIAPP_8-37_. (**a**,**b**) The representative plots showing the kinetics of (**a**) hIAPP_1-37_ and (**b**) hIAPP_8-37_ aggregation in the absence and presence of PEG-PE micelles. 20 µM solutions of hIAPP_1-37_ and hIAPP_8-37_ were incubated at 37 °C for 2 h with and without PEG-PE micelles at various PEG-PE/hIAPP molar ratios: 1:1 (20 µM/20 µM) and 10:1 (200 µM/20 µM). Error bars represent the standard deviation (*n* = 3). (**c,d**) TEM images of (**c**) hIAPP_1-37_ and (**d**) hIAPP_8-37_ after 24 h of incubation at 37 °C in the absence and presence of one and tenfold excess of PEG-PE micelles. The concentrations of hIAPP_1-37_ and hIAPP_8-37_ were 20 µM. (**e,f,g**) ^1^H-NMR spectra of (**e**) PEG-PE copolymer (5 mM), (**f**) hIAPP_1-37_ (0.5 mM) and (**g**) hIAPP_1-37_/PEG-PE (PEG-PE:hIAPP_1-37_ = 10:1, molar ratio) in D_2_O.

ThT binding assay is a widely used method for monitoring amyloid aggregation^24^. ThT can generate a new fluorescence excitation maximum at 450 nm and an enhanced emission at 482 nm upon binding with β-sheet structures of amyloid aggregates. To determine whether PEG-PE micelle can act as an inhibitor for both hIAPP_1__-__37_ and hIAPP_8__-__37_ aggregation, the aggregation kinetics of hIAPP_1__-__37_ and hIAPP_8__-__37_ were monitored by measuring ThT fluorescence intensity. The freshly prepared solutions of hIAPP_1-37_ and hIAPP_8-37_ were incubated in the absence and presence of PEG-PE micelles at different molar ratios (hIAPP:PEG-PE molar ratio was varied from 1:0 to 1:10), and the fluorescence emission of ThT was followed for 2 h. The relative ThT fluorescence intensity was plotted as a function of time. As shown in Fig. 1a, in the absence of PEG-PE micelles, the ThT fluorescence of hIAPP_1-37_ gradually increased after a lag phase of ≈15 min until it reached a plateau at ≈60 min, exhibiting a characteristic quasi-sigmoidal shape. Meanwhile, the ThT fluorescence of hIAPP_8-37_ reached a plateau at ≈30 min, implying that all of the hIAPP_8-37_ ended up in the form of β-sheet-rich amyloid (Fig. 1b). These results of ThT binding assay indicated that the aggregation of both hIAPP_1-37_ and hIAPP_8-37_ were consistent with a typical nucleation-growth mode^25^. In the presence of PEG-PE micelles, we first checked the ThT fluorescence of PEG-PE micelles alone (20 µM and 200 µM), and the result clearly demonstrated that the PEG-PE micelle didn’t react with ThT molecules (Fig. 1a,b). The ThT fluorescence of both hIAPP_1-37_ and hIAPP_8-37_ significantly decreased to ≈50% at 1:1 molar ratio of hIAPP:PEG-PE, and to ≈20% at 1:10 molar ratio of hIAPP:PEG-PE (Fig. 1a,b). The inhibitory effect of PEG-PE micelles on hIAPP_1-37_ and hIAPP_8-37_ aggregation was dose-dependent, indicated from the decreased ThT fluorescence with increasing concentrations of PEG-PE micelles. These results confirmed the strong suppression effect of PEG-PE micelles on both hIAPP_1-37_ and hIAPP_8-37_ fibrillogenesis.

To further verify the antiamyloidogenic activity of PEG-PE micelles on hIAPP_1-37_ and hIAPP_8-37_ aggregation, transmission electron microscope (TEM) was employed to detect the morphology of both hIAPP_1-37_ and hIAPP_8-37_ in the absence and presence of PEG-PE micelles. We found that, in the absence of PEG-PE micelles, both hIAPP_1-37_ and hIAPP_8-37_ formed typical mature fibrils with a length of several micrometers after 24 h of incubation at 37 °C (Fig. 1c,d). This observation was consistent with the previous reported studies that hIAPP can gradually self-assemble into larger aggregates, including oligomers and protofibrils, and finally form mature fibrils^26–29^. In the presence of PEG-PE micelles, the formation of mature fibrils of hIAPP_1-37_ and hIAPP_8-37_ was greatly inhibited when the molar ratios of PEG-PE/hIAPP were increased from 1/1 to 10/1 (Fig. 1c,d). When the molar ratio of PEG-PE to hIAPP was 10, hIAPP_1-37_ and hIAPP_8-37_ fibrils were completely replaced by spherical particles that were very similar to the morphology of empty PEG-PE micelles (Fig. 1c). TEM results demonstrated that PEG-PE micelles could effectively inhibit the aggregation of both hIAPP_1-37_ and hIAPP_8-37_, in accordance with the results from ThT binding assay.

To detect in detail the interactions of hIAPP and PEG-PE, we performed the nuclear magnetic resonance (NMR) experiments. We first characterized the physical state and the chemical composition of PEG-PE using ^1^H-NMR spectra. The concentration of PEG-PE (5 mM) in the NMR samples in this study was much higher than the critical micelle concentration (CMC) of PEG-PE in water (10 µM)^19^, which imply that all of PEG-PE molecules in the samples are in a form of the micelle. The assigned proton resonances for chemical groups of PEG-PE were indicated in Fig. 1e. The signals of protons in 11-CH_2_, 12-CH_2_O, 13-CH_2_O and 14-CH_3_O of PEG chains were much narrower than those in 1-CH_3_, 2-CH_2_, 3-CH_2,_ and 4-CH_2_ of PE chains. These results indicated that, in PEG-PE micelle, PEG chains have higher mobility than PE chains. Thus, the extended PEG chains form a hydrophilic outer shell, while the hydrophobic PE chains form a hydrophobic inner core in aqueous media. hIAPP_1-37_ showed intrinsic specific peaks at 0.5–7.5 ppm (Fig. 1f). As shown in Fig. 1g, the ^1^H-NMR spectrum of hIAPP_1-37_/PEG-PE at 1:10 molar ratio of hIAPP:PEG-PE in D_2_O also suggested the formation of the core-shell structure in comparison to the ^1^H-NMR spectrum of empty PEG-PE micelles (Fig. 1e). However, in the presence of PEG-PE micelles, the majority of specific peaks of hIAPP_1-37_ molecules at 1.0–4.5 ppm disappeared, and only the specific peaks at 7.0–7.2 ppm (black arrow 1*) and 0.6–0.9 ppm (black arrow 2*) still retained (Fig. 1g). Compared with hIAPP_1-37_, the resonances of hIAPP_1-37_ in the presence of PEG-PE micelles were significantly reduced and showed lower peaks that close to the baseline because of the decreased molecular motion of hIAPP_1-37_ encapsulated in the micelles. Therefore, the NMR results prove that hIAPP could be intercalated into PEG-PE micelles.

In addition, dynamic light scattering (DLS) was also employed to detect the particle size distribution of hIAPP_1-37_ and hIAPP_8-37_ aggregates in the absence and presence of PEG-PE micelles. These results clearly showed that the average diameters of hIAPP_1-37_ and hIAPP_8-37_ aggregates were 4.8 µm and 2.7 µm after 24 h of incubation at 37 °C, indicating the formation of hIAPP fibrils (Supplementary Figure S1a,b). However, after addition of PEG-PE, bimodal heterogeneous distribution was observed at a molar ratio of 1:1 (PEG-PE:hIAPP) as shown in Figure S1c, d. For hIAPP_1-37_/PEG-PE system, 93% of the particles have an average diameter of 28.9 nm ± 5.9 nm and 7% of the particles have an average diameter of 4.3 µm ± 1.5 µm) (Supplementary Figure S1c). The larger particles with diameter of about 4 µm could be the hIAPP aggregates. Similarly, for hIAPP_8-37_/PEG-PE system, 85% of the particles have an average diameter of 22.8 nm ± 6.0 nm and 15% of the particles have an average diameter of 0.9 µm ± 0.6 µm (Supplementary Figure S1d). Moreover, the particle size distribution was more homogenous and stable with a single peak at 19.8 nm ± 4.1 nm and 19.0 nm ± 4.2 nm at a molar ratio of 1:10 (hIAPP:PEG-PE) (Supplementary Figure S1e,f). These results indicated that the ability of PEG-PE micelles to attenuate hIAPP aggregation could also manifest in reduction of hIAPP aggregates size. The particle size of PEG-PE micelle alone is about 15.9 nm ± 3.8 nm and the increased particle size for PEG-PE/hIAPP system could be ascribed to the formation of hIAPP/PEG-PE complexes (Supplementary Figure S1g).

### PEG-PE micelle-induced conformational changes of hIAPP_1-37_ and hIAPP_8-37_

**Figure 2:**
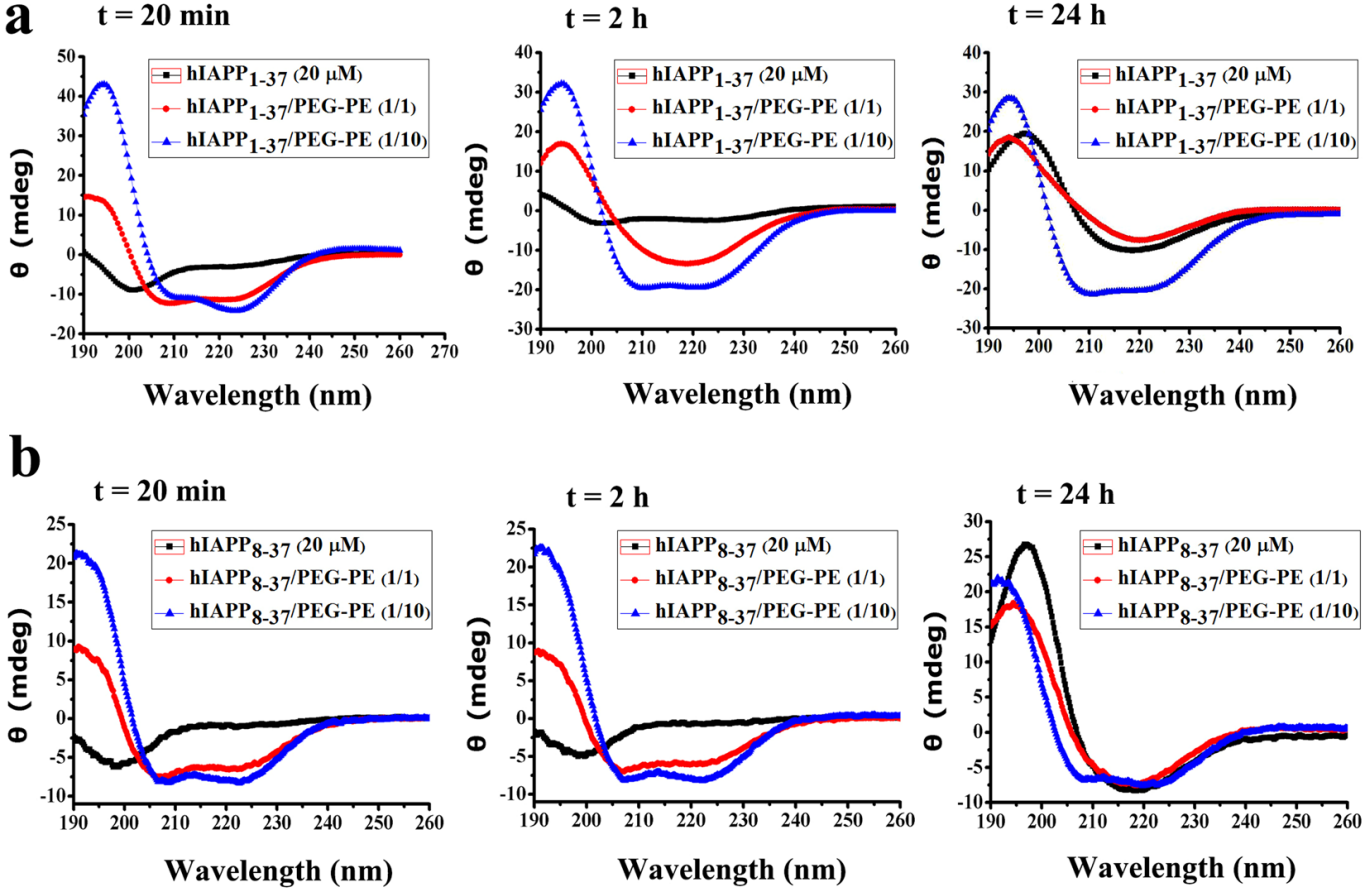
Circular dichroism spectra showing PEG-PE micelles inhibited conformational changes of hIAPP_1-37_ and hIAPP_8-37_. 20 µM solutions of (**a**) hIAPP_1-37_ and (**b**) hIAPP_8-37_ were incubated at 37 °C in the absence and presence of one and tenfold excess of PEG-PE micelles and analyzed after 20 min, 2 h and 24 h. The spectra represent the average of six scans after subtracting the contribution of the PEG-PE micelles at identical concentrations.

hIAPP aggregation is usually accompanied by a conformational change from the non-pathogenic random coils to the pathogenic β-sheet-rich structures^5^. To investigate the effect of PEG-PE micelles on the conformational change of hIAPP, we performed circular dichroism (CD) spectroscopy with hIAPP_1-37_ and hIAPP_8-37_ which were incubated in the absence and presence of one and tenfold excess concentrations of PEG-PE micelles for 20 min, 2 h and 24 h. As the introduction of PEG-PE micelles didn’t cause the conformational changes compared with PBS or ddH_2_O, the CD spectra of samples were measured on the baseline of PEG-PE micelles with identical concentration and incubation time. Within 2 h of incubation at 37 °C, both hIAPP_1-37_ and hIAPP_8-37_ in the absence of PEG-PE micelles retained their native random coil conformation with a negative band around 199 nm (Fig. 2a,b). After 24 h of incubation, both hIAPP_1-37_ and hIAPP_8-37_ exhibited a spectrum with a positive peak at around 195 nm and a negative peak at around 217 nm, characteristic of a β-sheet-rich structure (Fig. 2a,b). However, influenced by PEG-PE micelles, the ellipticity of the characteristic peak of β-sheet structures of both hIAPP_1-37_ and hIAPP_8-37_ decreased at 195 nm and increased at 208 nm and 222 nm in a dose-dependent manner (Fig. 2a,b). In addition, the proportions of secondary structures elements of hIAPP_1-37_ and hIAPP_8-37_ in the absence and presence of PEG-PE micelles were analyzed after 24 h of incubation at 37 °C. Compared with hIAPP_1-37_, PEG-PE micelles significantly altered the relative proportions of the secondary structure elements of hIAPP_1-37_ at a molar ratio of 10:1 (PEG-PE:hIAPP_1-37_): β-sheet contents decreased from 72.5% to 26.1%, α-helix contents increased from 0% to 32.8%, and random coil contents increased from 5.9% to 31.9%, respectively (Supplementary Table S1). Meanwhile, the relative proportions of secondary structure elements of hIAPP_8-37_ were also changed in the presence of tenfold excess concentration of PEG-PE micelles for 24 h as compared to hIAPP_8-37_ alone: β-sheet contents decreased from 61.4% to 31.2%, α-helix contents increased from 0% to 30.9%, and random coil contents increased from 0% to 29.2%, respectively (Supplementary Table S2). The CD results suggested that PEG-PE micelles induced a decrease in the content of β-sheet structures and an increase in the contents of α-helical and random coil structures of both hIAPP_1-37_ and hIAPP_8-37_, which supported the results from ThT binding assay. Since increased β-sheet constituent is the main cause of hIAPP aggregation^25^, PEG-PE micelles could hinder the conformational transition of hIAPP_1-37_ and hIAPP_8-37_ from random coil to β-sheet, which eventually inhibited the fibrils formation of hIAPP_1-37_ and hIAPP_8-37_.

### The inhibitory effect of PEG-PE micelles on hIAPP_1-37_ and hIAPP_8-37_ oligomerization and fibrillation

**Figure 3:**
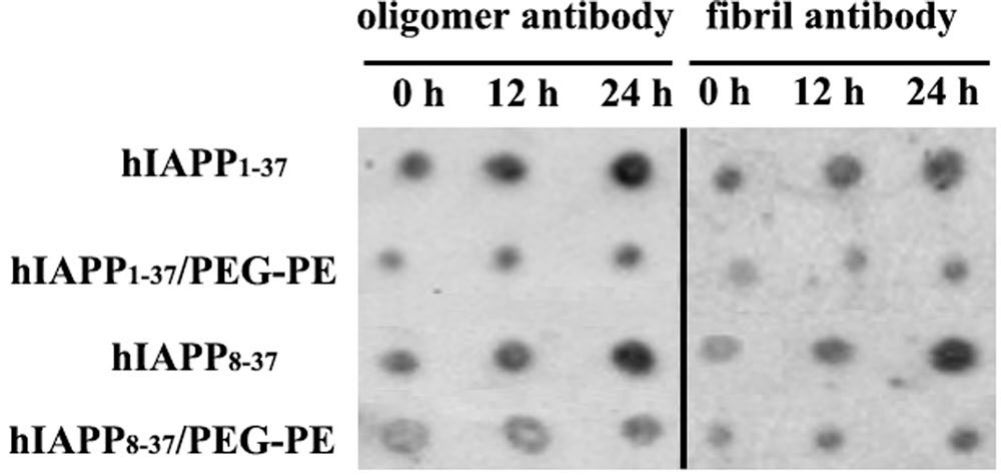
The effect of PEG-PE micelles on hIAPP_1-37_ and hIAPP_8-37_ oligomerization and fibrillation. Dot blots of 20 µM solutions of hIAPP_1-37_ and hIAPP_8-37_ were aged for 0, 12, 24 h in the absence and presence of 200 µM of PEG-PE micelles, spotted onto the nitrocellulose membranes, and probed with the anti-oligomer polyclonal antibody (A11). The same membranes of hIAPP_1-37_ and hIAPP_8-37_ in the absence and presence of 200 µM of PEG-PE micelles were also immunostained with the anti-amyloid fibrils polyclonal antibody.

Taken together, the ThT binding assay, TEM characterization, NMR spectroscopy, DLS characterization and CD spectroscopy showed that PEG-PE micelles inhibited hIAPP_1-37_ and hIAPP_8-37_ aggregation through inhibiting the β-sheet structures formation (Figs 1 and 2, Supplementary Figure S1, and Supplementary Tables S1, S2). Since PEG-PE micelles have amphiphilic nano-cages that consist of hydrophilic PEG chain and hydrophobic PE core as previously reported^21^, we hypothesized that PEG-PE micelles could effectively prevent hIAPP_1-37_ and hIAPP_8-37_ aggregation by capturing hIAPP_1-37_ and hIAPP_8-37_ monomers and its intermediate oligomeric aggregates into their nano-cages. In order to verify the hypothesis, the time-resolved dot blot assay was exploited to measure the formation of hIAPP_1-37_ and hIAPP_8-37_ oligomers and fibrils in the absence and presence of PEG-PE micelles. hIAPP_1-37_ and hIAPP_8-37_ were incubated in the absence and presence of tenfold excess amount of PEG-PE micelles for 24 h, and the abundance of both oligomers and fibrils at different time intervals were determined using anti-oligomer (A11) and anti-amyloid fibrils polyclonal antibodies^30,31^. As shown in Fig. 3, dot blot assay revealed that the amount of hIAPP_1-37_ and hIAPP_8-37_ oligomers and fibrils increased in a time-dependent manner in the absence of PEG-PE micelles, while significantly decreased in the presence of PEG-PE micelles and remained less over 24 h of incubation at 37 °C.

### The effect of PEG-PE micelles on hIAPP_1-37_ and hIAPP_8-37_ induced-cytotoxicity

**Figure 4:**
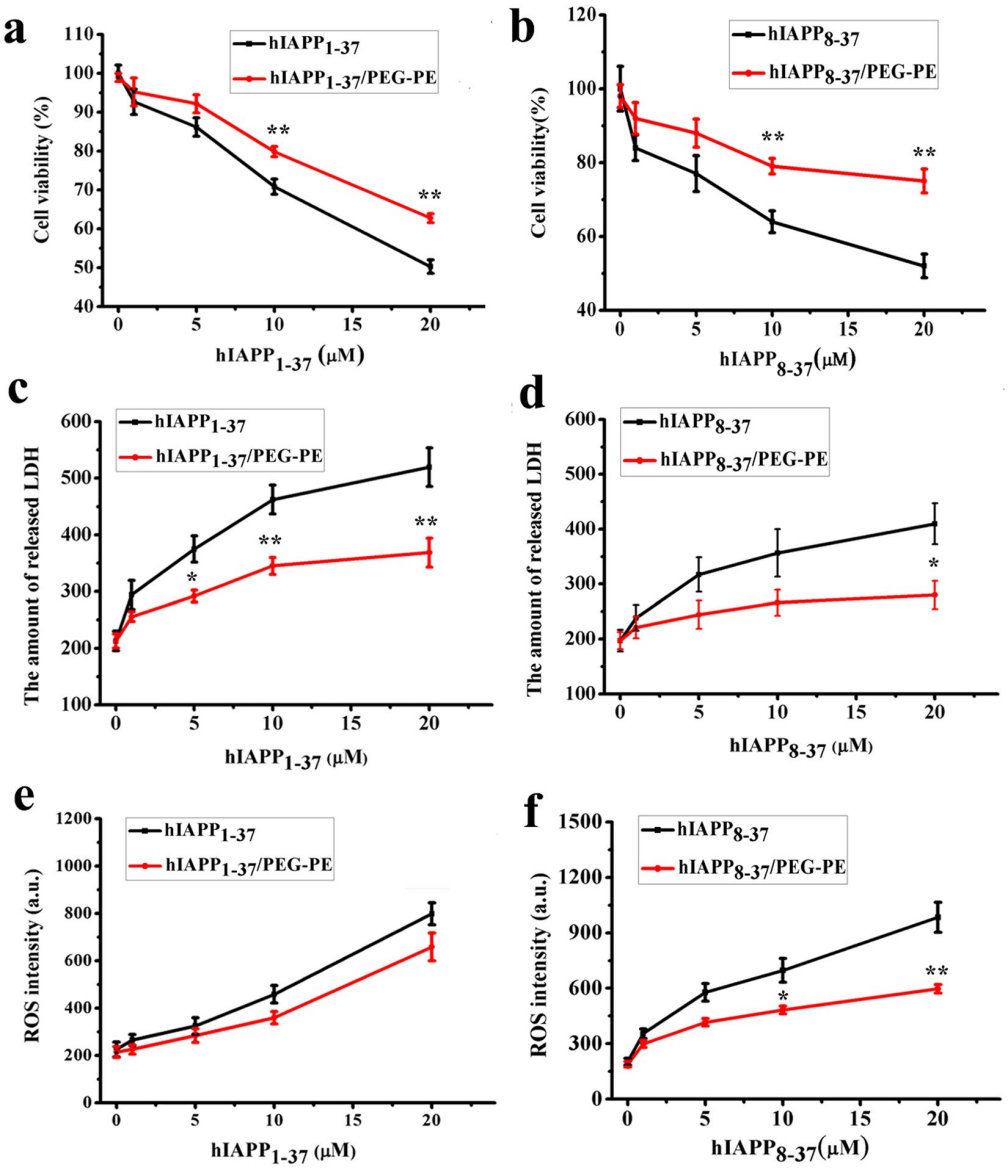
The effect of PEG-PE micelles on hIAPP_1-37_ and hIAPP_8-37_ induced-cytotoxicity. (**a**,**c**,**e**) hIAPP_1-37_ (1 µM, 5 µM, 10 µM, and 20 µM) and (**b,d,f**) hIAPP_8-37_ (1 µM, 5 µM, 10 µM, and 20 µM) solutions and the mixture of hIAPP_1-37_/PEG-PE (1 µM/20 µM, 5 µM/20 µM, 10 µM/20 µM, and 20 µM/20 µM) and hIAPP_8-37_/PEG-PE (1 µM/20 µM, 5 µM/20 µM, 10 µM/20 µM, and 20 µM/20 µM) were incubated with INS-1 cells for 24 and 48 h at 37 °C. (**a,b**) Cell viability was evaluated by the MTS assay. (**c**,**d**) The amount of released LDH in the culture medium was determined by a LDH assay reagent. (**e,f**) ROS was determined by measuring the fluorescence intensity of an oxidation-sensitive fluorescein DCFH-DA. Significance (*p < 0.05 and **p < 0.01) was calculated relative to hIAPP_1-37_ and hIAPP_8-37_, respectively. Error bars represent standard deviation (*n* = 3).

Since hIAPP aggregation is associated with the pathogenesis of T2DM^3,4,9^, we hypothesized that PEG-PE micelles that effectively inhibited the oligomerization and fibrillation of hIAPP_1-37_ and hIAPP_8-37_ might reduce the cytotoxicity mediated by hIAPP_1-37_ and hIAPP_8-37_. In this study, INS-1 cell line was used as a pancreatic β-cell model system to evaluate the effect of PEG-PE micelles on hIAPP_1-37_- and hIAPP_8-37_-induced cytotoxicity. The negligible cytotoxicity induced by PEG-PE itself with varying concentrations from 1 μM to 60 μM was observed (cell viability > 95%), suggesting the great biocompatibility of PEG-PE (Supplementary Figure S2). Taking the cytotoxicity issue into consideration, we used PEG-PE concentration under 60 μM for the following cell experiments. INS-1 cells were incubated with hIAPP_1-37_ (1–20 μM) and hIAPP_8-37_ (1–20 μM) in the absence and presence of PEG-PE micelles (20 µM) for 48 h. MTS assay was performed to determine the cell viability. We found that the viabilities of both hIAPP_1-37_- and hIAPP_8-37_-treated cells in the absence of PEG-PE micelles were gradually decreased when the concentrations of hIAPP_1-37_ and hIAPP_8-37_ were increased from 1 μM to 20 μM (Fig. 4a,b), indicating the cytotoxicity induced by hIAPP. PEG-PE micelle alone is nearly non-toxic (Supplementary Figure S2) and can significantly reduce the toxicity of hIAPP_1-37_ (Fig. 4a) and hIAPP_8-37_ (Fig. 4b) by decreasing the amount of hIAPP oligomers and fibrils.

It has been generally accepted that amyloid oligomers have greater cytotoxicity due to their higher membrane permeability as compared to monomers and mature fibrils^32,33^. The amyloid oligomer-induced membrane permeability, which leads to the cell dysfunction and cell death, can be inhibited by the anti-oligomer antibody^34^ or amyloid aggregation inhibitors^35^. In addition to the cell membrane permeability, a large number of studies have demonstrated that the generation of reactive oxygen species (ROS) during oxidative stress is also strongly linked to pancreatic β-cell death^36^. Treatment of β-cells with exogenous hIAPP resulted in intracellular ROS accumulation, and treatment with antioxidant inhibited the progression of hIAPP-induced β-cell death^37^. In order to clarify the underlying molecular mechanisms of inhibition of hIAPP-mediated β-cell death by PEG-PE micelle, we therefore evaluated the effect of PEG-PE micelles on hIAPP_1-37_- and hIAPP_8-37_-induced cell membrane permeability and oxidative stress. INS-1 cells were incubated with hIAPP_1-37_ (1–20 μM) and hIAPP_8-37_ (1–20 μM) in the absence and presence of PEG-PE micelles (20 µM) for 24 h. The results showed that the amount of released lactate dehydrogenase (LDH) was increased when the concentrations of hIAPP_1-37_ and hIAPP_8-37_ were increased from 1 μM to 20 μM in the absence of PEG-PE micelles (Fig. 4c,d), confirming the hIAPP_1-37_- and hIAPP_8-37_-induced cell membrane permeability. However, the amount of released LDH that induced by hIAPP_1-37_ and hIAPP_8-37_ in the presence of PEG-PE micelles is significantly decreased compared to hIAPP_1-37_ and hIAPP_8-37_ alone (Fig. 4c,d). Meanwhile, intracellular ROS was measured by a fluorescein-labeled dye DCFH-DA. Treatment with hIAPP_1-37_ and hIAPP_8-37_ alone increased the intracellular ROS level of INS-1 cells in a dose-dependent manner (Fig. 4e,f), confirming that the oxidative stress was also responsible for hIAPP_1-37_- and hIAPP_8-37_-induced cell death, which was consistent with the previous report^36,37^. In contrast, declined ROS level in hIAPP_8-37_-treated cells because of the addition of PEG-PE micelles was observed, suggesting that PEG-PE micelles effectively rescued hIAPP_8-37_-induced INS-1 cell death through alleviating the oxidative stress (Fig. 4f). Taken together, these results demonstrated that both the cell membrane permeability and the oxidative stress play central roles in hIAPP-mediated β-cell death. PEG-PE micelles were demonstrated to inhibit hIAPP_1-37_ and hIAPP_8-37_ oligomerization and fibrillation and to rescue hIAPP_1-37_- and hIAPP_8-37_-induced cytotoxicity. PEG-PE micelles reduced the cytotoxicity by decreasing the cell membrane permeability and alleviating the oxidative stress.

### PEG-PE micelles can remodel the preformed hIAPP_1-37_ and hIAPP_8-37_ fibrils and rescue fibrils-mediated cytotoxicity to INS-1 cells

**Figure 5:**
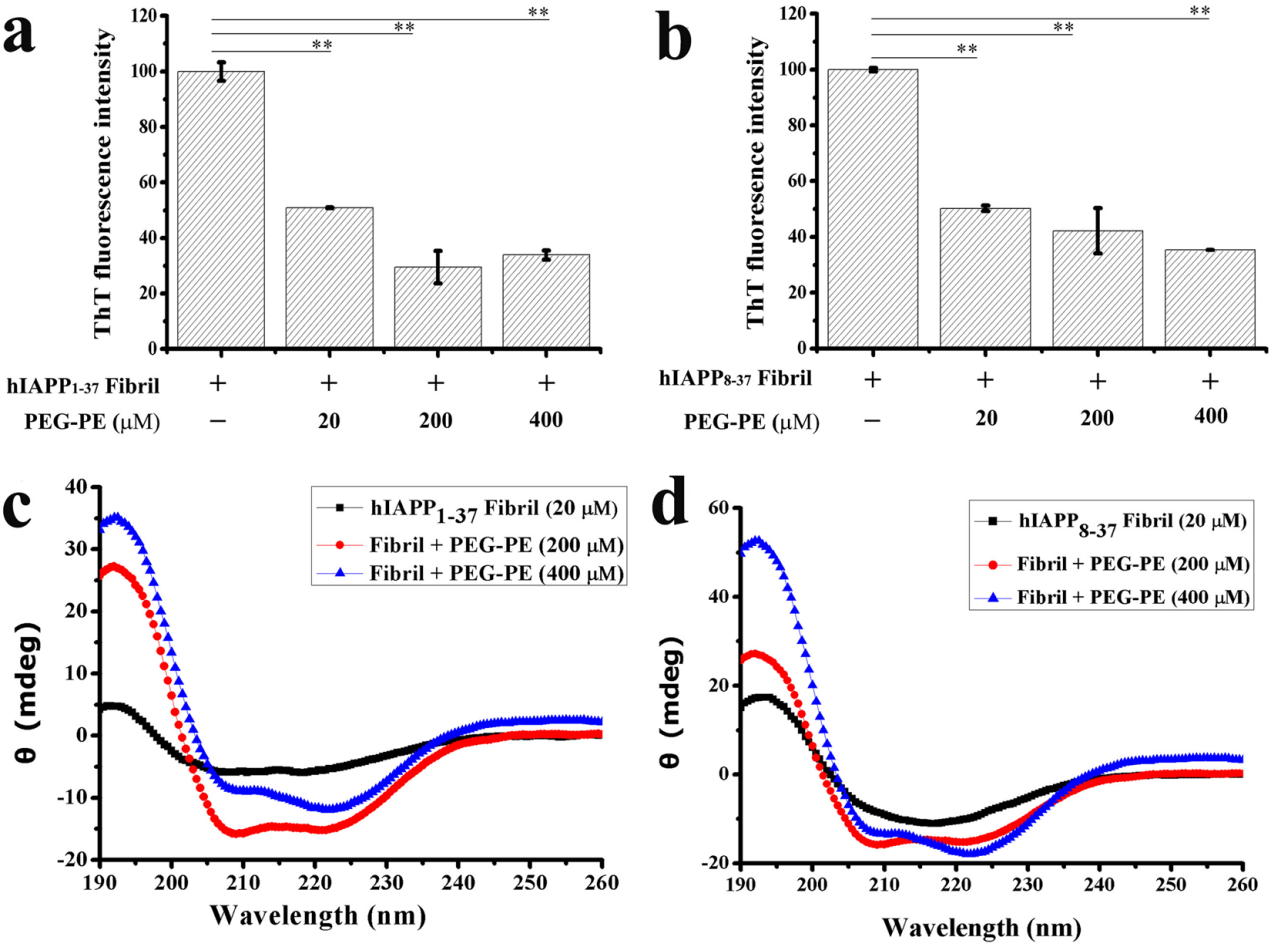
PEG-PE micelles can reverse the conformational transition of hIAPP_1-37_ and hIAPP_8-37_ fibrils from β-sheet to α-helical structures. (**a**,**b**) 20 μM solutions of (**a**) hIAPP_1-37_ and (**b**) hIAPP_8-37_ were aged for 24 h at 37 °C to afford maximal ThT fluorescence. The aged samples were then incubated with freshly prepared PEG-PE micelles (20 μM, 200 μM, and 400 μM) for an additional 96 h and then examined by ThT binding assay. The ThT fluorescence values of aged hIAPP_1-37_ fibril and hIAPP_8-37_ fibril in the absence of PEG-PE micelles were referred to as 100%. Significance (*p < 0.05 and **p < 0.01) was calculated relative to the hIAPP_1-37_ and hIAPP_8-37_ fibrils, respectively. Error bars represent standard deviation (*n* = 3). (**c**,**d**) CD spectra of (**c**) hIAPP_1-37_ (20 μM) and (**d**) hIAPP_8-37_ (20 μM) were aged for 24 h at 37 °C, and the aged samples were incubated with and without tenfold and twentyfold excess concentrations of PEG-PE micelles for an additional 96 h. The spectra represent the average of six scans after subtracting the contribution of the PEG-PE micelles at identical concentrations.

Since the extracellular aggregation of hIAPP and the fibrils extension can cause the invagination and perforation of cell membranes^36^, inhibitors that have an ability to remodel the preformed mature fibrils are desired for rescuing mature fibrils-mediated cytotoxicity. In our previous work, we have demonstrated that, in the presence of DTT, PEG-PE micelles not only prevented insulin aggregation but also remodelled the preformed insulin aggregates^20^. Therefore, we further investigated whether this property of PEG-PE micelles is applicable to the preformed hIAPP fibrils. 20 μM solutions of hIAPP_1-37_ and hIAPP_8-37_ were aged for 24 h at 37 °C to form fibrils and then exposed to the increasing concentrations of PEG-PE micelles for a further 96 h. The aged hIAPP_1-37_ and hIAPP_8-37_ fibrils in the absence of PEG-PE micelles exhibited a pronounced ThT signal, but the addition of PEG-PE micelles reduced the signal in a dose-dependent manner (Fig. 5a,b). PEG-PE micelles concentration higher than/or equal to stoichiometric ratio can effectively remodel hIAPP fibril. Results indicated that PEG-PE micelles actively remodelled the relatively stable 1:1 co-aggregates formation through reacting with monomers or small oligomers of hIAPP_1-37_ and hIAPP_8-37_. PEG-PE micelles shifted the hIAPP_1-37_ and hIAPP_8-37_ aggregation equilibrium toward monomers and small oligomers, which allowed the assembly of these co-aggregates. Furthermore, we determined the conformational changes of the aged samples of hIAPP_1-37_ and hIAPP_8-37_ using CD spectra, samples were incubated with and without PEG-PE micelles for an additional 96 h before analysis. hIAPP_1-37_ and hIAPP_8-37_ fibrils mainly adopted a predominant β-sheet structures in the absence of PEG-PE micelles (Fig. 5c,d). The secondary structures of hIAPP_1-37_ and hIAPP_8-37_ fibrils were significantly changed from β-sheet into α-helix in the presence of 10-fold and 20-fold excess amount of PEG-PE micelles, which supported the results from ThT binding assay.

**Figure 6:**
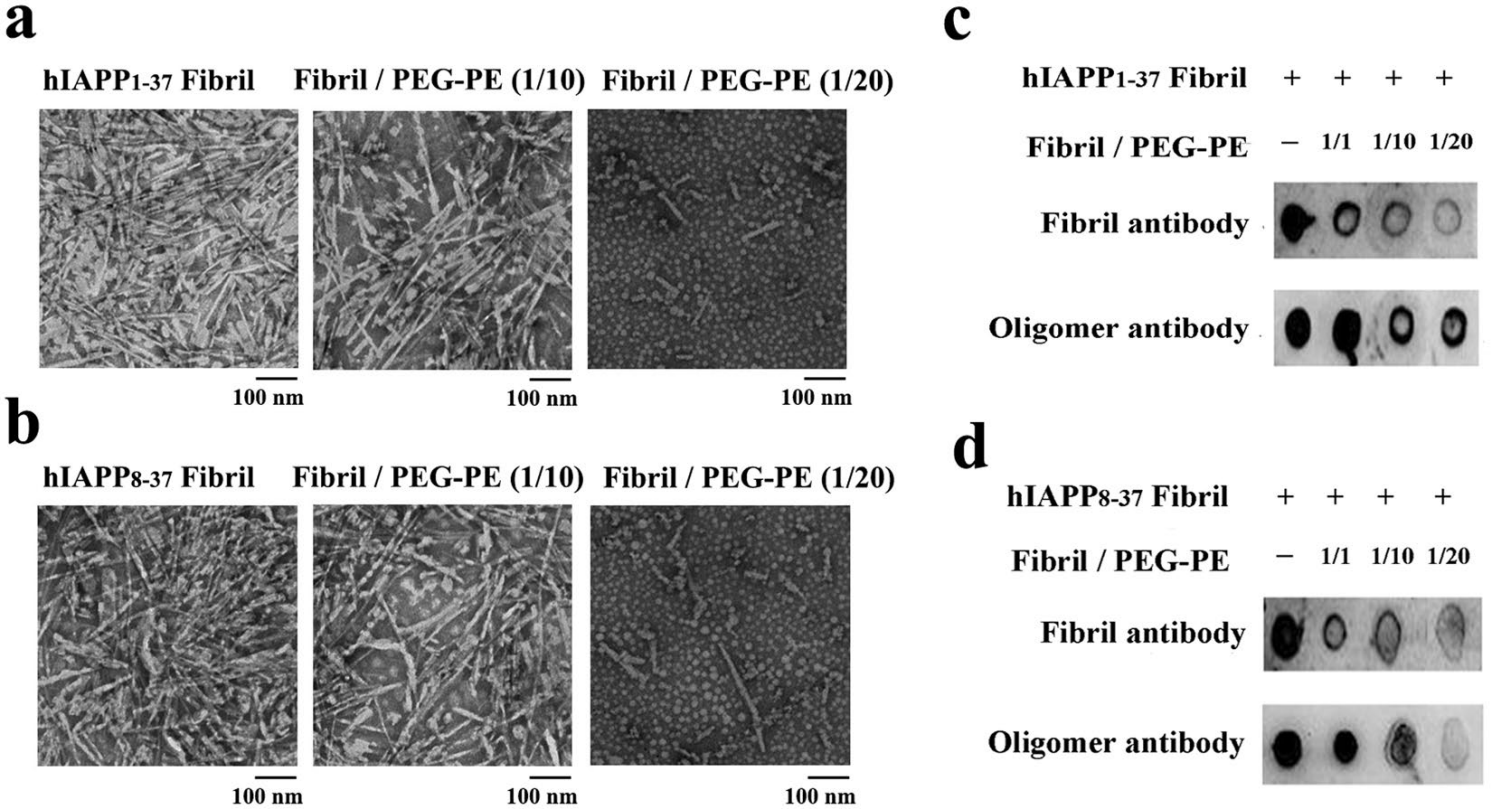
PEG-PE micelles can remodel fibrillar hIAPP_1-37_ and hIAPP_8-37_ into species that are non-reactive toward oligomer- and fibril antibodies. (**a**,**b**) TEM images of (**a**) hIAPP_1-37_ (20 μM) and (**b**) hIAPP_8-37_ (20 μM) were aged for 24 h at 37 °C, and the aged samples were incubated with and without tenfold and twentyfold excess concentrations of PEG-PE micelles for an additional 96 h. (**c**,**d**) Dot blots of 20 μM of (**c**) hIAPP_1-37_ and (**d**) hIAPP_8-37_ were aged for 24 h at 37 °C, and the aged samples were then incubated with and without freshly prepared PEG-PE micelles (20 μM, 200 μM, and 400 μM) for a further 96 h. The disaggregation products were tested by dot blot assay using anti-oligomer and anti-amyloid fibril polyclonal antibodies.

The remodelling of fibrillar hIAPP_1-37_ and hIAPP_8-37_ by PEG-PE micelles was also confirmed by TEM analysis. Samples of hIAPP_1-37_ and hIAPP_8-37_ fibrils in the absence and presence of PEG-PE micelles were loaded over copper grids directly after CD experiments. Both hIAPP_1-37_ and hIAPP_8-37_ formed long unbranched fibrils as they aged, but PEG-PE micelles significantly decreased the amount of hIAPP_1-37_ and hIAPP_8-37_ fibrils in a dose-dependent manner (Fig. 6a,b). Compared with hIAPP_1-37_ and hIAPP_8-37_ fibrils, the average length of fibrils in the presence of PEG-PE micelle was shortened, but the average width of fibrils did not change.

**Figure 7:**
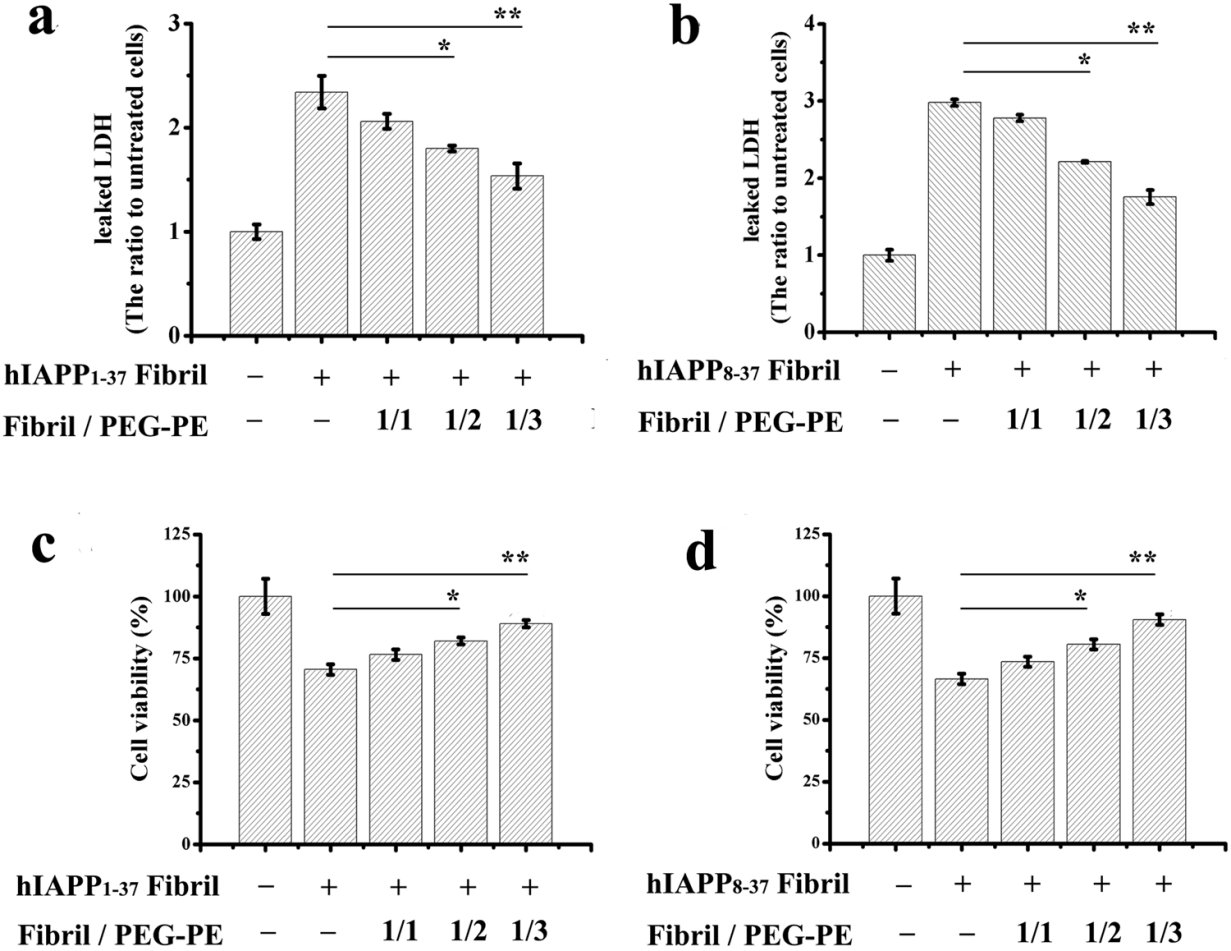
Dose-dependent effect of PEG-PE micelles on the hIAPP_1-37_ and hIAPP_8-37_ fibrils-mediated cytotoxicity to INS-1 cells. (**a**,**c**) hIAPP_1-37_ (20 μM) and (**b**,**d**) hIAPP_8-37_ (20 μM) were aged for 24 h at 37 °C, and the aged samples were then incubated for a further 96 h in the absence and presence of increasing concentrations of PEG-PE micelles (20 μM, 40 μM, and 60 μM). hIAPP_1-37_ and hIAPP_8-37_ fibrils and the mixture of fibril/PEG-PE were exposed to INS-1 cells for an additional 24 and 48 h. (**a**,**b**) The amount of released LDH in the culture medium was determined by a LDH assay reagent. (**c**,**d**) The cell viability was measured by the MTS assay. Results were expressed as a percentage of the control group and were reported as mean ± standard deviation (SD) from three assays. Significance (*p < 0.05 and **p < 0.01) was calculated relative to the hIAPP_1-37_ and hIAPP_8-37_ fibrils, respectively.

The remodelling of hIAPP_1-37_ and hIAPP_8-37_ fibrils by PEG-PE micelles could possibly produce adverse effects *in vivo* if it increases the amount of toxic intermediates, such as amyloid oligomers^32,33^. In order to confirm that the remodelling of hIAPP fibrils does not involve the formation of toxic soluble oligomers, PEG-PE micelles-treated hIAPP_1-37_ and hIAPP_8-37_ fibrils were tested for their reactivity toward the anti-oligomer and anti-amyloid fibril antibodies. The results demonstrated that PEG-PE micelles effectively decreased the amounts of both oligomers and amyloid fibrils of the aged hIAPP_1-37_ and hIAPP_8-37_ samples in a dose-dependent manner (Fig. 6c,d), implying that the remodelling of hIAPP_1-37_ and hIAPP_8-37_ fibrils by PEG-PE micelles led to the formation of co-aggregates that were distinct from soluble oligomers and mature fibrils. Next, we determined whether PEG-PE micelles could reduce hIAPP fibrils-induced cytotoxicity to INS-1 cells. hIAPP_1-37_ and hIAPP_8-37_ fibrils and the mixture of fibrils/PEG-PE were incubated with INS-1 cells for an additional 24 and 48 h. Amount of released LDH and cell viability were evaluated according to the above-mentioned procedures. The results from MTS assay indicated that hIAPP fibrils-induced cytotoxicity was attenuated by PEG-PE micelles dose-dependently (Fig. 7c,d). In accordance with MTS results, declined LDH release due to the addition of PEG-PE micelles further validated the reduced hIAPP fibrils-mediated cytotoxicity (Fig. 7a,b).

**Figure 8:**
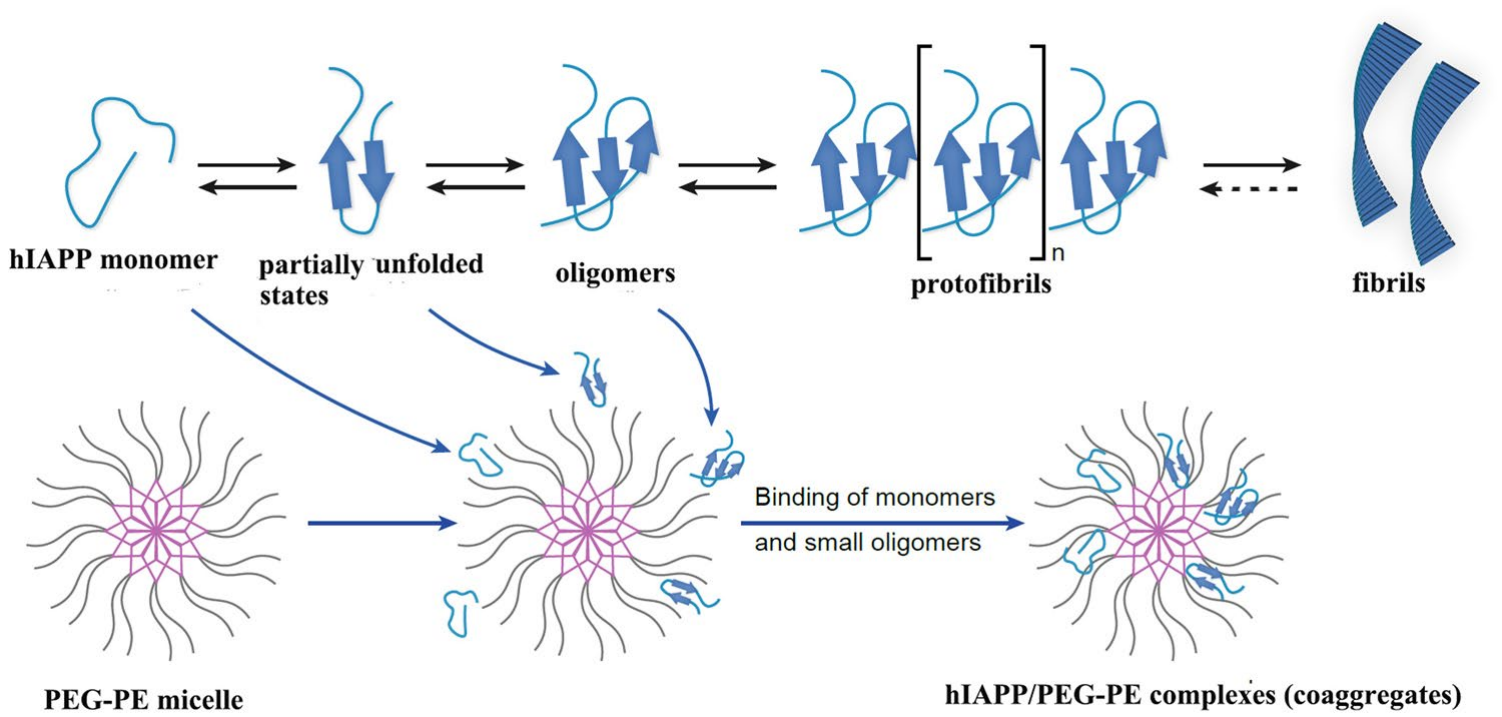
Schematic illustration of the possible mechanism of the interaction between PEG-PE micelles and hIAPP on the kinetics of amyloid fibrillation process.

As shown in Fig. 8, the soluble monomeric form of hIAPP peptide undergoes specific conformational transitions into aggregation-prone “partially unfolded intermediates” and subsequently aggregates into oligomers. The oligomers serve as templates for further hIAPP deposition, resulting in rapid fibril growth and eventually in the formation of insoluble amyloid fibrils, as previously reported^38^. Although the precise mechanism that how PEG-PE micelle inhibits hIAPP aggregation and remodels the preformed hIAPP fibrils required further research. A plausible mechanism may be proposed that, in the presence of PEG-PE micelles, the partially unfolded state of the monomers and other small intermediate units of hIAPP_1-37_ and hIAPP_8-37_ were encapsulated into the amphiphilic nano-cages of PEG-PE micelles, which led to the formation of hIAPP/PEG-PE complexes (co-aggregates). This process could block active sites hidden in the hIAPP amyloidogenic domain that is responsible for its aggregation. Meanwhile, the hydrophilic PEG chains could create a protective barrier layer, which prevents the excessive adsorption of hIAPP_1-37_ and hIAPP_8-37_.

Moreover, the addition of PEG-PE micelles was able to decrease the monomeric concentrations of hIAPP_1-37_ and hIAPP_8-37_ in solution, which disturbed the dynamic equilibrium between monomeric and oligomeric species and shifted the hIAPP aggregation pathway from an “on-pathway” to an “off-pathway” mechanism.

## Discussion

Although insulin can temporarily alleviate the conditions of T2DM, but currently there is no effective treatment that can suppress or cure this disease. In addition, evidences from clinical studies suggest that people suffering from T2DM have greater chance of developing AD in comparison to healthy individuals^39^, hence researches for new therapeutic strategies for T2DM are still important and in demand. It has been widely recognized that the aggregation of monomeric hIAPP into insoluble plaque-associated amyloid fibrils is a crucial step that drives T2DM pathogenesis^4,38^. Based on this hypothesis regarding amyloids, extensive efforts have been dedicated to developing hIAPP inhibitors^11–18^. Among developed hIAPP inhibitors, nanomaterials have been drawing great attention due to their small size and physicochemical properties, especially their partial hydrophobicity. This property of nanomaterials allows them to pass through cell membranes and enter different cells and organelles, perturbing self-assembly pathways of proteins^15^. Either inhibitory or enhancement effect on the process of amyloid aggregation have been reported, while the application of the nanomaterials in remodelling amyloid aggregation is quite limited due to their biocompatibility issues^40,41^. Therefore, it is necessary and of great significance to develop nanomaterials with good safety and biocompatibility as a novel hIAPP inhibitor, so that more therapeutic options can be provided to the patients.

In this work, we demonstrated that self-assembled PEG-PE micelles effectively rescued hIAPP_1-37_- and hIAPP_8-37_-induced cytotoxicity through either inhibiting hIAPP_1-37_ and hIAPP_8-37_ oligomerization and fibrillation or remodelling the preformed hIAPP_1-37_ and hIAPP_8-37_ fibrils. PEG-PE copolymer that consists of hydrophilic PEG and hydrophobic PE is ideal for forming micelles, and PEG-PE micelles have a low CMC (~10 µM) and small size distribution (~15-20 nm)^19^. Moreover, PEG-PE micelles show superior biocompatibility as they were approved by the Food and Drug Administration (FDA) as a pharmaceutical excipient for Doxil^®^ (liposomal formulation of doxorubicin)^42^.

In this study, the inhibitory effects of PEG-PE micelles on hIAPP_1-37_ and hIAPP_8-37_ are very similar. There are two possible reasons to explain this phenomenon. One reason could be related to the isoelectric points (PI) of hIAPP_1-37_ (PI:8.9), hIAPP_8-37_ (PI:8.8) and PEG-PE polymer (PI:5.93). At pH 7.4, This PEG-PE polymer is negatively charged due to the de-protonation of the phosphate group that exists between hydrophilic PEG and hydrophobic PE. Both hIAPP_1-37_ and hIAPP_8-37_ are positively charged, and they have almost the same charge due to their similar PI. The electrostatic interactions between these two peptides and PEG-PE micelles may play critical roles in hIAPP encapsulation, which inhibited hIAPP aggregation. The other reason is that the first 7 amino acids have neglectable impact on the aggregation of hIAPP. The β-hairpin structure of hIAPP has been obtained by solid-state NMR, suggesting the parallel β-sheet structures with 10 residues in the core domains^43^. The fragment IAPP_1-7_ is believed to be non-β-sheet structure as the result of the disulfide bond between cysteine residues 2 and 7^44^. Thus these two peptides have similarity in β-sheet structures and also aggregation propensity.

Extensive studies have shown that the imbalance between hIAPP anabolism and catabolism in the pancreatic islet is the primary event responsible for the pathogenesis of T2DM^38^. In healthy individuals, the production of hIAPP is normally in equilibrium with its excretion from body. Both overproduction and imbalance in excretion from body could disrupt hIAPP homeostasis, which leads to dysfunction and death of pancreatic β-cell due to the generation of hIAPP amyloid aggregates with variable cytotoxicity. From preceding results it is proved that PEG-PE micelles possess capability to maintain hIAPP homeostasis by simultaneously inhibiting hIAPP aggregation and promoting disaggregation of hIAPP aggregates that eventually diminished hIAPP-mediated cytotoxicity. In conclusion, this study may provide an attractive therapeutic strategy for generating a PEG-PE micelle-based anti-diabetic agent that will help to develop new drugs for T2DM therapy.

## Methods

### Chemicals and materials

Lyophilized powders of hIAPP_1-37_ and hIAPP_8-37_ were purchased from GL Biochem Ltd. (Shanghai, China), and the purity of peptides (>98%) has been verified by high performance liquid chromatography (HPLC) and mass spectrum (MS). Distearoyl-*sn*-glycero-3-phosphoethanolamine-*n*-[methoxy (polyethylene glycol)-2000] (PEG-PE) was purchased from Avanti Polar Lipids (Alabaster, AL). INS-1 cell line was purchased from Chinese Academy of Medical Science & Peking Union Medical College, Beijing, China. All other chemicals were of analytical grade and used without any further purification.

### hIAPP and hIAPP/PEG-PE preparation

hIAPP_1-37_ and hIAPP_8-37_ preparations were prepared according to the previously published protocol with modification^28^. Briefly, hIAPP_1-37_ and hIAPP_8-37_ were dissolved in HFIP to the final concentration of 1 mM and aliquoted in microcentrifuge tubes. The solutions were sonicated in a water bath for 10 min and centrifuged at 4 °C for 10 min. The supernatants of hIAPP_1-37_ and hIAPP_8-37_ were collected and stored at −80 °C. Prior to use, HFIP was evaporated by vacuum rotary evaporator, and hIAPP_1-37_ and hIAPP_8-37_ solutions were prepared by dissolving with PBS and ddH_2_O using DMSO as a hydrotropic agent. On the other hand, hIAPP_1-37_ and hIAPP_8-37_ encapsulated into PEG-PE micelles were prepared via the film dispersion method, as previously described^19^. Firstly, the supernatant of hIAPP_1-37_ and hIAPP_8-37_ solutions in HFIP was mixed with a PEG-PE solution in chloroform at different molar ratios of 1:1~20 (hIAPP:PEG-PE) at room temperature. Secondly, the organic solvent of the mixed solution was evaporated by vacuum rotary evaporator to form a dry lipid film. Finally, the lipid film was rehydrated with PBS and ddH_2_O to obtain the hIAPP_1-37_ and hIAPP_8-37_ encapsulated into PEG-PE micelles (hIAPP_1-37_/PEG-PE and hIAPP_8-37_/PEG-PE).

### ThT binding assay

The extent of hIAPP_1-37_ and hIAPP_8-37_ aggregation in the absence and presence of PEG-PE micelle was determined using ThT (a fluorescent dye that specifically binds with the β-sheet of amyloid structures^24^). hIAPP_1-37_ and hIAPP_8-37_ solutions (20 μM in PBS) and the mixture of hIAPP_1-37_/PEG-PE and hIAPP_8-37_/PEG-PE (20 μM/20 μM, and 20 μM/200 μM) were added into the wells of 96-well plates. Each sample has three replicated wells and each well contains 20 μM ThT. The plates were covered with Platemax CyclerSeal sealing film (Axygen, USA) and incubated at 37 °C for 2 h to follow hIAPP aggregation dynamics. The fluorescence intensity of each well was measured by spectrophotometer (SpectraMax i3, Molecular Devices, USA) at the excitation and emission wavelengths of 450 and 482 nm, respectively.

### TEM characterization

20 μM solutions of hIAPP_1-37_ and hIAPP_8-37_ in ddH_2_O in the absence and presence of 20 μM and 200 μM PEG-PE micelles were incubated for 24 h at 37 °C. 10 µL of each sample was dropped over the copper grid and allowed to adsorb for 10 min at room temperature. After that, the excessive solution was removed from the copper grid by the filter paper. Samples were stained with 1% uranyl acetate for 1 min and washed three times with ddH_2_O. TEM measurements were performed with HITACHI TEM (Hitachi, Ltd., Tokyo, Japan) with 80 kV accelerated voltage.

### NMR Spectroscopy

PEG-PE stock solution (10 mM) was prepared by dissolving the powder in deuterium oxide. hIAPP_1-37_ stock solution (1 mM) was prepared by dissolving the powder in deuterium oxide using DMSO-d6 as a hydrotropic agent. hIAPP_1-37_ encapsulated into PEG-PE micelles (hIAPP_1-37_/PEG-PE, hIAPP_1-37_: PEG-PE = 1:10, molar ratio) were prepared via the film dispersion method, and the lipid film was rehydrated with deuterium oxide. All NMR spectra were acquired at 298 K with Bruker DPX 400 spectrometer equipped with a Z-gradient BBO probe. The ^1^H NMR experiments were performed on the samples containing 0.5 mM hIAPP_1-37_ or 5 mM PEG-PE in D_2_O.

### DLS characterization

20 μM solutions of hIAPP_1-37_ and hIAPP_8-37_ in ddH_2_O in the absence and presence of 20 μM and 200 μM PEG-PE micelles were incubated for 24 h at 37 °C.

Particle size distribution of each sample was determined by DLS analysis using Nano Particle Analyzer (Malvern Instrument Ltd, Malvern, UK).

### CD spectroscopy

CD measurements were performed using Jasco-J810 spectrometer (Jasco, Tokyo, Japan) under a constant flow of nitrogen gas. Freshly prepared solutions of hIAPP_1-37_ and hIAPP_8-37_ in PBS (20 μM) were incubated and analyzed in the absence and presence of 20 μM and 200 μM PEG-PE micelles for 20 min, 2 h and 24 h. Measurements were performed at room temperature in a fused quartz cuvette (1-mm path length). CD spectra were recorded in the range of 190 to 260 nm with a step size and a bandwidth of 1 nm. For each CD measurement, 6 runs were averaged and corrected by subtracting the contribution of the PEG-PE at identical concentrations.

### Dot Blot assay

Freshly prepared solutions of hIAPP_1-37_ and hIAPP_8-37_ (20 μM) were incubated at 37 °C for over 24 h in the absence and presence of PEG-PE micelles (200 μM). Samples were collected at 0, 12, 24 h time intervals, respectively. Later on 10 μL of each sample was dropped over a nitrocellulose membrane (0.22 μm) and dried at room temperature. The membrane was blocked with 5% (w/v) nonfat milk in tris-buffered saline containing 0.1% Tween 20 (TBST) for 1 h at room temperature. After being washed with TBST for three times, the membrane was incubated with the anti-oligomer polyclonal antibody (A11) and anti-amyloid fibrils polyclonal antibody at 1:1000 dilution in 5% (w/v) nonfat milk in TBST overnight at 4 °C. Then the membrane was incubated with horseradish peroxidase (HRP) conjugated anti-rabbit IgG at 1:1000 dilution in 5% (w/v) nonfat milk in TBST for 1 h at room temperature. After being washed with TBST for three times, the membrane was developed with enhanced chemiluminescence system.

### Cell viability assay

The cell viability was measured using the CellTiter 96^®^ Aqueous One Solution Cell Proliferation assay (also termed as MTS assay, Promega, Madison, Wisconsin, USA). INS-1 cells were plated at 8,000 cells per well in the 96-well plates and were grown overnight before replacing the medium with freshly prepared hIAPP_1-37_ and hIAPP_8-37_ solutions (1 μM, 5 μM, 10 μM, and 20 μM) and with the mixture of hIAPP_1-37_/PEG-PE and hIAPP_8-37_/PEG-PE (1 μM/20 μM, 5 μM/20 μM, 10 μM/20 μM, and 20 μM/20 μM). After 48 h, 20 μL of MTS reagent was added to each well followed by incubation for another 2 h at 37 °C. The absorbance was measured at 490 nm using spectrophotometer. Wells with culture medium alone (no cells) served as blank controls, and the value of the control cells that treated with same volume of corresponding solution in the absence of hIAPP/PEG-PE was set as 100%.

### LDH assay

The release of LDH indicates the change of cell membrane permeability, which can reflect the extent of cell membranes damage^45^. INS-1 cells were plated at 8,000 cells per well in the 96-well plates and were grown overnight. Later on, cell were treated with freshly prepared hIAPP_1-37_ and hIAPP_8-37_ solutions (1 μM, 5 μM, 10 μM, and 20 μM) in the absence and presence of PEG-PE micelles (20 μM) followed by incubation for 24 h at 37 °C. After 24 h, the cell culture medium was transferred to a new 96-well plate followed by the addition of 50 μL of LDH assay reagent (Promega, Madison, Wisconsin, USA) into each well. Later on, cells were incubated in the dark for 30 min at room temperature, and the absorbance of each well was measured at 490 nm using spectrophotometer. The control cells were treated with same volume of corresponding solution in the absence of hIAPP/PEG-PE.

### ROS assay

50,000 cells/well were plated in 24-well plates and cultured overnight. Later on cells were exposed to freshly prepared hIAPP_1-37_ and hIAPP_8-37_ solutions (1 μM, 5 μM, 10 μM, and 20 μM) and to the mixture of hIAPP_1-37_/PEG-PE and hIAPP_8-37_/PEG-PE (1 μM/20 μM, 5 μM/20 μM, 10 μM/20 μM, and 20 μM/20 μM) followed by incubation for an additional 24 h at 37 °C. After incubation period of 24 h, cell culture medium of each well was replaced with 1 mL fresh medium containing 1 μL of ROS probe DCFH-DA (10 mM). After 20 min of incubation at 37 °C with DCFH-DA, 1 × 10^4^ cells were collected and subjected to Accuri^TM^ C6 flow cytometer (BD Biosciences, San Jose, CA), acquired data was analysed with CellQuest software.

### Fibrils remodelling assay

hIAPP_1-37_ and hIAPP_8-37_ solutions were incubated in ddH_2_O for 24 h at 37 °C to produce hIAPP_1-37_ and hIAPP_8-37_ fibrils. Later on, the aged hIAPP_1-37_ and hIAPP_8-37_ fibrils were mixed with PEG-PE micelles at different molar ratios from 1:1 to 20:1 (PEG-PE: Fibril) followed by incubation for an additional 96 h at 37 °C. The ThT binding assay, CD spectroscopy, TEM, dot blot assay, LDH assay and cell viability assay of hIAPP_1-37_ and hIAPP_8-37_ fibrils in the absence and presence of PEG-PE micelles were performed according to the above-mentioned experimental procedure.

### Statistical analysis

All experiments were carried out at least three times with three independent samples. Data were expressed as means ± SD unless noted otherwise. Unpaired Student’s t-test was performed to assess the statistical significance of the results and was expressed in terms of *p* values, *p* value less than 0.05 are indicated by * and *p* value less than 0.01 are indicated by **.

## Electronic supplementary material

Supplementary Information
