# Supplementary material for: Dual effect of PEG-PE micelle over the oligomerization and fibrillation of human islet amyloid polypeptide
Source: Sci Rep. 2018 Mar 13;8:4463. doi: 10.1038/s41598-018-22820-w (PMC5849606; doi:10.1038/s41598-018-22820-w)
Supplement: Supplementary file 1 — Supplementary Information [file 41598_2018_22820_MOESM1_ESM.pdf]

Dual effect of PEG-PE micelle over the oligomerization and fibrillation of human islet amyloid polypeptide

Xiaocui Fang[1], Maryam Yousaf[1,2], Qunxing Huang[1], Yanlian Yang[1],*, Chen Wang[1],*

[1]CAS Key Laboratory of Standardization and Measurement for Nanotechnology, CAS Key Laboratory of Biological Effects of Nanomaterials and Nanosafety, CAS Center for Excellence in Nanoscience, National Center for Nanoscience and Technology, Beijing 100190, P. R. China.

[2]Radiation Chemistry Laboratory, Department of Chemistry, University of Agriculture, Faisalabad, 38000, Pakistan.

* Correspondence and requests for materials should be addressed to Y.Y. (yangyl@nanoctr.cn) or C.W. (wangch@nanoctr.cn)

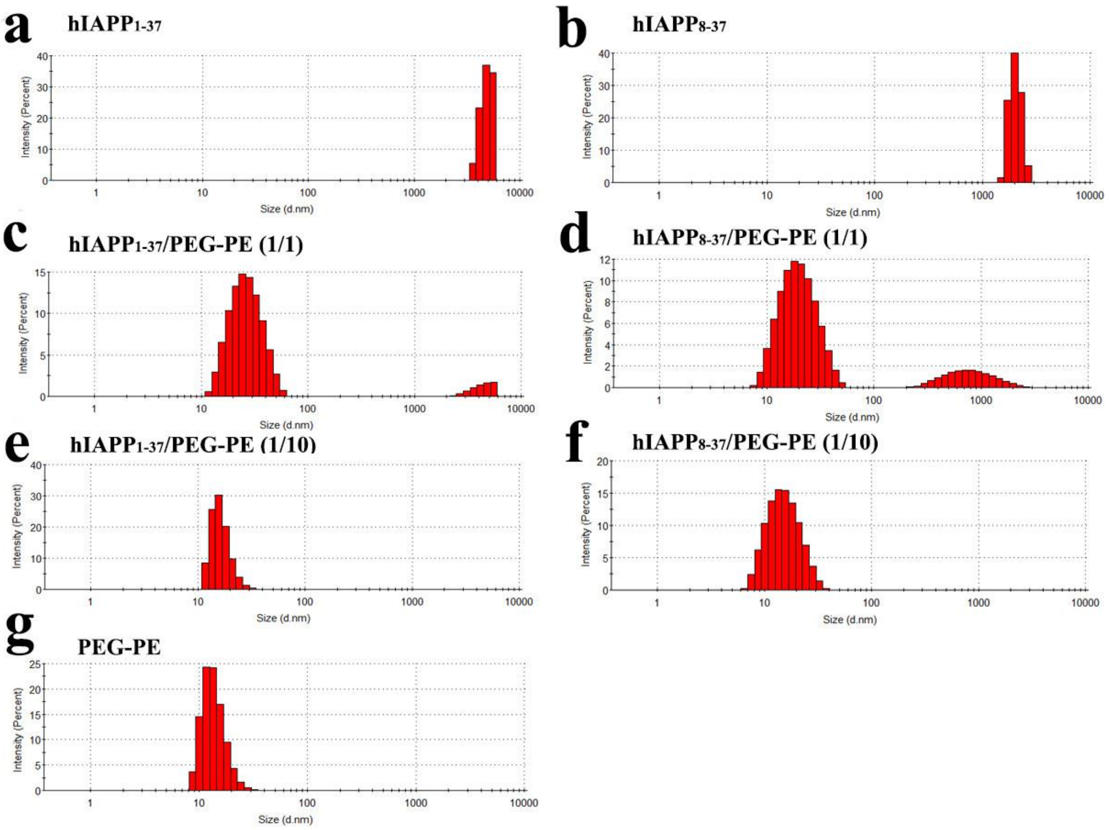

Figure S1. Particle size distribution of hIAPP$_{1-37}$ (20 μM) and hIAPP$_{8-37}$ (20 μM) aggregates in the absence and presence of PEG-PE micelles (20 μM and 200 μM) after 24 h of incubation at 37 ℃.

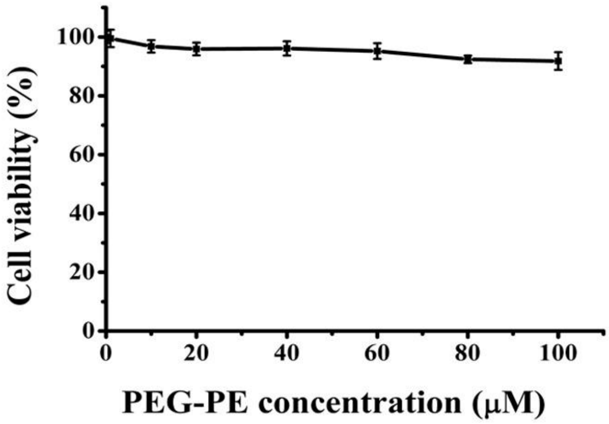

Figure S2. *In vitro* cytotoxicity assay of INS-1 cells, assessed by MTS, after incubation with PEG-PE (0-100 μM) for 48 h at 37 ℃.

Table S1. Percentages of secondary structure elements of $hIAPP_{1-37}$ with different treatments

		**α-helix**	**β-sheet**	**β-turn**	**random coil**
t = 20 min	$hIAPP_{1-37}$	0.2% ± 0.4%	27.1% ± 1.3%	23.0% ± 3.6%	49.7% ± 5.1%
	$hIAPP_{1-37}$/PEG-PE (1/1)	29.0% ± 2.9%	26.1% ± 0.6%	11.5% ± 1.3%	33.4% ± 3.2%
	$hIAPP_{1-37}$/PEG-PE (1/10)	48.4% ± 4.2%	19.0% ± 3.2%	1.8% ± 0.4%	30.8% ± 1.9%
t = 2 h	$hIAPP_{1-37}$	0.3% ± 0.2%	30.2% ± 3.6%	18.5% ± 4.6%	51.0% ± 4.2%
	$hIAPP_{1-37}$/PEG-PE (1/1)	39.0% ± 6.3%	36.1% ± 1.9%	8.2% ± 0.9%	16.7% ± 3.1%
	$hIAPP_{1-37}$/PEG-PE (1/10)	59.8% ± 5.2%	10.8% ± 1.3%	3.6% ± 0.1%	25.8% ± 2.5%
t = 24 h	$hIAPP_{1-37}$	0.0% ± 0.1%	72.5% ± 5.4%	21.6% ± 2.9%	5.9% ± 0.6%
	$hIAPP_{1-37}$/PEG-PE (1/1)	10.1% ± 2.5%	49.2% ± 3.4%	29.1% ± 2.7%	11.6% ± 1.8%
	$hIAPP_{1-37}$/PEG-PE (1/10)	32.8% ± 1.9%	26.1% ± 3.9%	9.2% ± 1.7%	31.9% ± 1.4%

Table S2. Percentages of secondary structure elements of $hIAPP_{8-37}$ with different treatments

		**α-helix**	**β-sheet**	**β-turn**	**random coil**
t = 20 min	$hIAPP_{8-37}$	0.0% ± 0.5%	23.3% ± 2.1%	18.2% ± 4.7%	58.4% ± 6.3%
	$hIAPP_{8-37}$/PEG-PE (1/1)	33.2% ± 3.1%	35.2% ± 3.6%	0.0% ± 0.7%	31.6% ± 4.2%
	$hIAPP_{8-37}$/PEG-PE (1/10)	54.3% ± 5.8%	19.9% ± 6.3%	0.0% ± 0.4%	25.8% ± 2.5%
t = 2 h	$hIAPP_{8-37}$	0.0% ± 0.7%	20.6% ± 2.3%	18.7% ± 3.1%	60.8% ± 5.6%
	$hIAPP_{8-37}$/PEG-PE (1/1)	37.8% ± 0.9%	31.7% ± 2.4%	0.0% ± 1.1%	30.5% ± 2.5%
	$hIAPP_{8-37}$/PEG-PE (1/10)	65.8% ± 6.0%	9.5% ± 0.9%	0.0% ± 0.8%	24.8% ± 4.8%
t = 24 h	$hIAPP_{8-37}$	0.0% ± 1.2%	61.4% ± 6.8%	38.6% ± 3.6%	0.0% ± 0.7%
	$hIAPP_{8-37}$/PEG-PE (1/1)	16.2% ± 2.1%	39.2% ± 1.9%	21.6% ± 1.6%	23.0% ± 1.5%
	$hIAPP_{8-37}$/PEG-PE (1/10)	30.9% ± 2.1%	31.2% ± 4.7%	8.6% ± 1.2%	29.2% ± 2.9%
